# Is serum albumin a pivotal biomarker in anticipating acute pancreatitis outcomes?

**DOI:** 10.1186/s12876-024-03314-8

**Published:** 2024-07-24

**Authors:** Fakhrddine Amri, Maissae Rahaoui, Hanane Aissaoui, Ouiam Elmqaddem, Hajar Koulali, Abdelkrim Zazour, Naima Abda, Zahi Ismaili, Ghizlane Kharrasse

**Affiliations:** 1 Department of Hepato-Gastroenterology, Mohammed VI University Hospital, Oujda, Morocco; 2 Digestive Diseases Research Laboratory (DSRL), Faculty of Medicine and Pharmacy, Mohammed First University, Oujda, Morocco; 3 Laboratory of Epidemiology, Clinical Research, and Public Health, Faculty of Medicine and Pharmacy, Mohammed First University, Oujda, Morocco

**Keywords:** Acute Pancreatitis, Serum albumin, Hypoalbuminemia, Prognosis, Severity scores, Mortality, Clinical outcomes

## Abstract

This study aimed to assess the significance of serum albumin levels within 24 h of patient admission in correlation with the incidence of outcomes and mortality in patients diagnosed with acute pancreatitis. A retrospective study was conducted over a 5-year period, from January 2018 to December 2023, at the Mohammed VI University Hospital in Oujda, Morocco. The study included 371 patients diagnosed with acute pancreatitis. Hypoalbuminemia (≤ 30 g/L) was observed in 124 patients (33.4% of cases), and these patients had a higher mean age compared to those with normal albumin levels (*P* = 0.003). Hypoalbuminemia was significantly associated with persistent Systemic Inflammatory Response Syndrome (SIRS) (70.8% vs. 29.2%, *P* = 0.000), a higher BISAP score (66.7% vs. 33.3%, *P* = 0.000), and a higher CTSI score (51.7% vs. 48.3%, *P* = 0.000). Hypoalbuminemia was also associated with the presence of pleural effusion (*P* = 0.000). The mortality in the sample was 4.6%, and it was significantly associated with hypoalbuminemia (76.5%, *P* = 0.000). In conclusion, serum albumin levels within 24 h of patient admission appear to be a significant prognostic biomarker in acute pancreatitis, particularly in anticipating persistent organ failure and mortality.

## Introduction

Acute pancreatitis (AP) is an inflammation of the pancreas with a worldwide incidence ranging from 30 to 40 cases per 100,000 population per year [1]. It’s usually caused by bile stones or excessive alcohol consumption. Most patients experience mild symptoms, which can be managed with moderate fluid resuscitation, pain and nausea management, and early oral feeding. However, some cases may require hospitalization and intensive care due to the development of local or systemic complications, including organ failure. Severe AP is redefined as AP with persistent organ failure lasting more than 48 h, with a mortality rate ranging from 20 to 50%, as per the 2012 revised Atlanta classification for AP [2]. Several factors have been associated with severity in AP, notably smoking, dyslipidemia, obesity, pancreatic necrosis, and pancreatic collections [3].

Albumin is the most abundant protein in the body and serves a variety of essential functions. These include the regulation of oncotic pressure, as well as the binding and transportation of both endogenous and exogenous substances. Additionally, albumin plays a crucial role in antioxidant functions [4].

Hypoalbuminemia is frequently observed in various clinical conditions, such as sepsis, kidney failure, nephrotic syndrome, cancer, decompensated liver cirrhosis, surgical procedures. This phenomenon can also manifest in acute pancreatitis, particularly in cases of severe AP [5].

Some studies have shown that low serum albumin levels in AP are significantly related to poor prognosis, and could be an important tool for predicting adverse outcomes, especially in anticipating persistent organ failure and mortality [5].

Our study aimed to assess the significance of serum albumin levels within 24 h of patient admission in correlation with the incidence of outcomes and mortality in AP.

## Materials and methods

### Design of the study and population

This is a retrospective study conducted over a 5-year period, from January 2018 to December 2023, at the Mohammed VI University Hospital in Oujda, Morocco. The research question was defined prior to the commencement of the study, focusing on the significance of serum albumin levels within 24 h of hospital admission in patients diagnosed with AP.

### Diagnosis and classification of AP

The diagnostic align with the 2012 revision of the Atlanta classification, requiring the presence of two or more of the following three criteria [6]:

1. Abdominal pain consistent with AP.
2. Imaging findings from contrast-enhanced computed tomography (CT), magnetic resonance imaging (MRI), or abdominal ultrasonography characteristic of AP.
3. Serum amylase and/or lipase elevation ≥ three times the upper limit of normal.

### Exclusion criteria comprised the following

1. Patients younger than 16 years old.
2. Unavailable laboratory measurements or medical records.
3. Conditions with an underlying pathology that could affect serum albumin levels, including but not limited to malabsorption syndromes, chronic pancreatitis, liver cirrhosis, chronic renal disease, albuminuria, nephrotic syndrome, severe malnutrition, and inflammatory bowel disease. At our center, routine checks for albuminuria are conducted as part of the comprehensive evaluation for patients with acute pancreatitis.

### Cutoff for hypoalbuminemia

A cutoff value of 30 g/L for albumin levels was employed for grouping, and this threshold aligns with values used in prior studies [7, 8].

### Scoring systems employed to evaluate severity (Table 1):

**Table 1:** Bedside Index of Severity in Acute Pancreatitis (BISAP) [9]:

Parameters	Score 0	Score 1
Blood urea nitrogen	< 25 mg/dl	> 25 mg/dl
Impaired mental status	Absent	Present
SIRS	Absent	Present
Age	< 60 years	> 60 years
Pleural effusion	Absent	Present

- **Systemic Inflammatory Response Syndrome (SIRS) : diagnosed by presence of any two of criteria**:

1. Temperature (< 36c or > 38c),
2. Pulse > 90/min,
3. Respiratory Rate > 20 or PaCO2 < 32mmHg, and.
4. WBC > 12,000/mm3 or < 4,000/mm3 or > 10% bands.

- **Bedside Index of Severity in Acute Pancreatitis (BISAP)** [9].
- **CT Severity Index (CTSI)** [10] **(**Table 2**)**:

**Table 2:** CT severity index (CTSI) [10]:

CT grade	Grade score	Definition
A	0	Normal pancreas
B	1	Pancreatic enlargement
C	2	Pancreatic inflammation and/or peripancreatic fat
D	3	Single peripancreatic fluid collection
E	4	≥ 2 fluid collections and/or retroperitoneal air
% of necrosis	Necrosis score	Definition
None	0	Uniform pancreatic enhancement
< 30%	2	Non-enhancement of region(s) of gland equivalent in size of pancreatic head
30–50%	4	Non-enhancement of 30–50% of the gland
> 50%	6	Non-enhancement of over 50% of the gland
CT Severity Index	Morbidity	Mortality
0–1	0	0
2–3	8%	3%
4–6	35%	6%
7–10	92%	17%

### Statistical analysis

Continuous data were reported as means ± standard deviations or medians (interquartile ranges) and were subjected to analysis using the student’s t-test or Mann–Whitney U test, depending on the appropriateness of the distribution. Categorical data were represented as absolute numbers and percentages, and the chi-square test was employed for analysis. Variables exhibiting a significant association with hypoalbuminemia in the univariable models (*P* < 0.05) were subsequently included in the multivariable models to discern variables linked to albumin levels.

The odds ratio (OR) and corresponding 95% confidence intervals (CI) were calculated. A two-tailed *P* value < 0.05 was deemed statistically significant. All statistical analyses were conducted using SPSS software (v21.0).

## Results

A total of 371 patients were enrolled, with a notable female predominance of 70.9% compared to 29.1% for males, resulting in a male-to-female ratio of 2.43. The mean age was 55.5 years ± 18.699 (16–98 years). The percentage of diabetic patients was 11.3%. The primary etiology was biliary in 77.1%, post-ERCP (Endoscopic Retrograde Cholangiopancreatography) in 3%, drug-induced in 2.4%, hypertriglyceridemia in 1.9%, hypercalcemia in 1.3%, chronic alcohol-consumption in 1.6% of cases, with other etiologies accounting for 6.5%, and idiopathic pancreatitis observed in 6.2%. The average duration between symptom onset and consultation was 4.86 +/- 4.502 days. According to the NRI score, severe malnutrition was observed in 7% of patients. Anemia was observed in 18% of the patients.

On imaging, a CTSI score between 4 and 10 was observed in 40.2% of patients. Regarding clinical and laboratory scores of severity, 17.5% of cases exhibited persistent SIRS after 48 h, and 16.2% of cases had a BISAP score of ≥ 3. The in-hospital mortality rate was 4.6% (Table 3).

**Table 3:** Baseline characteristics of our patients

Variable	Number	Percent %
**Age**: mean & SD:	55.5 ± 18.6	
**Gender**		
Female Male	263 108	70.9 29.1
**Etiologies**		
Biliary Post-ERCP Drug-induced Hypertriglyceridemia Hypercalcemia Alcohol consumption Other Idiopathic	286 11 9 7 5 6 24 23	77.1 3 2.4 1.9 1.3 1.6 6.5 6.2
**Ascites**		
No Yes	326 45	87.9 12.1
**Pleural effusion** :		
No Yes	314 57	59.8 49.2
**CTSI (4–10 Pts)**		
No Yes	222 149	59.8 40.2
**BISAP score (3–5 Pts)**		
No Yes	311 60	83.8 16.2
**SIRS**		
Absent Present Persistent > 48 H	252 54 65	67.9 14.6 17.5
**Death**		
No Yes	354 17	95.4 4.6
**WON**		
No Yes	342 29	92.2 7.8
**Pseudocyst**		
No Yes	365 6	98.4 1.6
**Hypoalbuminemia** ≤ 30 g/L		
No Yes	247 124	66.6 33.4

The mean albumin level was 36.1 +/- 5.831 g/L (16–57), with hypoalbuminemia (≤ 30 g/L) observed in 124 patients, constituting 33.4% of cases. Patients with hypoalbuminemia had a higher mean age (59.62 ± 18.552) compared to those with normal albumin levels (53.54 ± 18.646) (*P* = 0.003). However, no significant differences were found regarding gender, BMI and the duration between symptoms onset and consultation.

In relation to vital signs, among patients exhibiting altered consciousness, 75.6% demonstrated hypoalbuminemia (*P* = 0.032). Hypoalbuminemia was also observed in all patients who experienced hypovolemic shock (*P* < 0.001). Furthermore, a significant association was identified between the presence of fever and hypoalbuminemia (*P* < 0.001).

Univariate analysis revealed also a significant association between low serum albumin levels and elevated mean creatinine, C-reactive protein (CRP) and urea levels. The mean creatinine level was 12.5 mg/l ± 19.333 in patients with hypoalbuminemia compared to 7.5 mg/l ± 4.298 in those with normal albumin levels (*P* = 0.006). Similarly, the mean CRP level was 180 mg/l ± 114.137 in patients with hypoalbuminemia compared to 116 mg/l ± 99.374 in those with normal albumin levels (*P* < 0.001). Concerning urea: (*p* < 0.001, mean difference of -0.18454, 95% CI [-0.27393, -0.09515]). A decline in serum albumin level was also associated with a decrease in hematocrit levels (*P* < 0.001). Patients with hypoalbuminemia also had a lower average hemoglobin level (11.7 ± 2.8 vs. 12.8 ± 1.7, *P* < 0.001). (Table 4). x.

**Table 4:** Baseline clinical characteristics and outcomes among patients with various albumin levels

	Normal 247 (66.6%)	Hypoalbuminemia 124 (33.4%)	*P* value
**Mean age**	53 ± 18	59 ± 18	0.003
**Gender**			
**Men** **Women**	66 (61.1) 181 (68.8)	42 (38.9) 82 (31.2)	0.183
**Consciousness disorder**	11 (24.4)	34 (75.6)	0.032
**hypovolemic shock**	0 (0)	7 (100)	< 0.001
**Fever**	19 (35.8)	34 (64.2)	< 0.001
**Ascites**	16 (35.6)	29 (64.4)	< 0.001
**Pleural effusion**	18 (31.6)	39 (68.4)	< 0.001
**SIRS > 48 H**	19 (29.2)	46 (70.8)	< 0.001
**BISAP ≥ 3**	20 (33.3)	40 (66.7)	< 0.001
**CTSI ≥ 4**	72 (48.3)	77 (51.7)	< 0.001
**Death**	4 (23.5)	13 (76.5)	< 0.001
**WON**	12 (41.4)	17 (58.6)	0.001
**Pseudocyst**	3 (50)	3 (50%)	0.315
**Evolution to chronic pancreatitis**	9 (69.2)	4 (30.8)	0.975
**Duration between symptoms and consultation (Mean**,** days)**	4.5 ± 4	5.5 ± 5	0.065
**BMI (Mean**,** Kg)**	24.4 ± 4	24.1 ± 4	0.509
**Creatinine (Mean**,** mg/l)**	7.5 ± 4	12.5 ± 19	0.006
**Urea (Mean**,** g/l)**	0.29 ± 0.29	0.48 ± 0.61	0.002
**CRP (Mean**,** mg/dl)**	116 ± 99	180 ± 114	< 0.001
**Hematocrit (Mean**,** %)**	38 ± 6	35 ± 5	< 0.001

Regarding severity scores: a significant association was found between hypoalbuminemia, SIRS, BISAP score, and CTSI. Among patients with persistent SIRS after 48 h, 70.8% had hypoalbuminemia, and 29.2% had normal albumin levels (*P* < 0.001). 66.7% of patients with a BISAP score ≥ 3 had hypoalbuminemia compared to 33.3% with normal albumin levels (*P* < 0.001). Among patients with a CTSI score > 3, 51.7% had hypoalbuminemia (*P* < 0.001). Hypoalbuminemia was also associated with the presence of pleural effusion (*P* < 0.001) and ascites (*P* < 0.001).

**Fig. 1:**
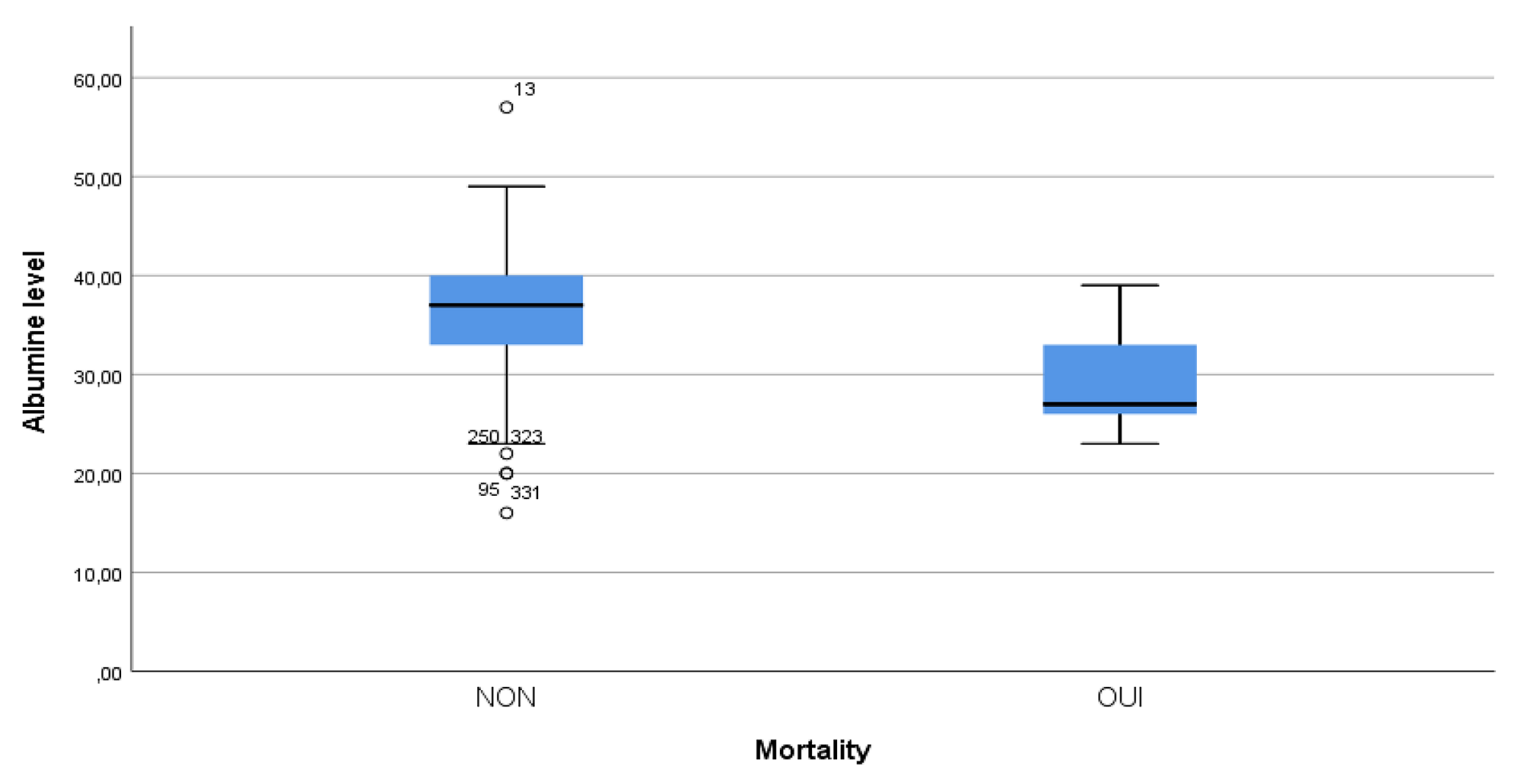
Examining Albumin Levels in Deceased and Living Patients

The mortality in our sample was 4.6%, and it was significantly associated with hypoalbuminemia (76.5%, *P* < 0.001). The mean albumin level in deceased patients was significantly lower compared to living patients (29.6 ± 5.7 vs. 36.4 ± 5.6 g/l, *P* < 0.001) (Fig. 1).

In terms of local complications, among patients who developed Walled-off Necrosis (WON), 58.6% exhibited hypoalbuminemia (*P* = 0.001); however, no significant association was found with the development of pseudocysts or infection of necrotic collections (Table 4).

During patient monitoring, among those who developed diabetes, 75% had hypoalbuminemia (*P* = 0.05). However, no significant association was found with the development of chronic pancreatitis, exocrine pancreatic insufficiency, and recurrence.

In the multivariate analysis, we included variables that showed a significant association with hypoalbuminemia in the univariate analysis. Subsequent steps in the logistic regression refinement process led to a final model that included the following variables: Age (*P* < 0.001, 95% CI [1.015, 1.045]), CRP (*P* = 0.03, 95% CI [1.003, 1.008]), CTSI (*P* < 0.001, 95% CI [1.588, 5.460]), SIRS (*P* < 0.001, 95% CI [1.782, 7.667]), and Hematocrit (*P* < 0.001, 95% CI [0.848, 0.930]). This final model suggests that these variables are statistically significant predictors of hypoalbuminemia in the studied population (Table 5).

**Table 5:** Binary logistic regression between albumin levels and the explanatory variables

	OR	95% confidence interval	*P*-value
Age	1.030	1.015–1.045	< 0.001
CRP	1.007	1.003–1.008	0.030
Hematocrit	0.888	0.848–0.930	< 0.001
CTSI	2.930	1.588–5.460	0.001
SIRS > 48 H	3.696	1.782–7.677	< 0.001

## Discussion

Human albumin is a small globular protein, primarily synthesized by the liver, with a molecular weight of 66.5 kilodaltons (kDa). It is the most abundant circulating protein found in the plasma (3.5 g/dL to 5 g/dL) of healthy individuals [11]. Functioning as the foremost modulator of plasma oncotic pressure, it serves as a carrier for various substances known as ligands. These ligands can be either endogenous, such as bilirubin, ions, and fatty acids, or exogenous, including drugs [11].

Serum albumin appears to be a reliable prognostic indicator in various contexts [12]. Studies suggest that serum albumin may serve as an independent predictor of mortality across a broad spectrum of clinical and research settings. It has been reported that an estimated increase in the risk of death ranges from 24 to 56% for every 2.5 g per liter decrease in serum albumin concentration across the studies examined [12].

Hypoalbuminemia has been identified in various inflammatory, infectious, and neoplastic pathologies [13]. Furthermore, it was independently linked to 30-day in-hospital mortality based on a retrospective analysis of data from over 20,000 emergency medical patients in Ireland [13]. Similarly, this phenomenon has also been observed in AP, particularly in patients with severe AP, and the percentage can reach one-third of patients, as observed in our study (33.4%), in contrast to the 19% reported by Klementina Ocskay and all. [14]. However, there are few studies that have investigated the prognostic impact of hypoalbuminemia in acute pancreatitis.

The mechanism underlying hypoalbuminemia in acute pancreatitis is complex and not fully elucidated. Some research proposes that it could be attributed to diminished protein intake resulting from pain-induced malnutrition. Additionally, increased catabolism of proteins induced by inflammatory cytokines, notably IL-6 and TNF-α, is considered a contributing factor. The active inflammatory cytokines also damage microvascular endothelial cells, leading to elevated capillary permeability. Consequently, this process results in the redistribution of albumin from the intravascular to the interstitial space [15, 16].

The effect of serum albumin levels on the clinical outcomes of AP has been the focus of investigation in a few reports. Gonzálvez-Gasch et al. reported that patients with low serum albumin levels had a higher risk of unfavorable outcomes, accelerating the deterioration of severe AP. This condition was associated with a higher incidence of infection and mortality. In their study, the sole criterion for hypoalbuminemia was a level < 25 g/L [17].

In a retrospective study conducted in Wuhan, Shoukang Li et al. concluded that serum albumin remained an independent prognostic factor for persistent organ failure (POF) in AP, with an OR of 0.748 (95% confidence interval: 0.645–0.868; *p* < 0.05 [2]. Additionally, in a prospective cohort study, patients with AP and multiorgan failure (MOF; *n* = 18) demonstrated a more pronounced decline in serum albumin levels (*P* < 0.001) compared to those without MOF [18].

Wenzheng Zhang et al. investigated the effectiveness of red cell distribution width (RDW), creatinine, and albumin in predicting AP mortality through ROC analysis. The results revealed that RDW had the highest sensitivity (88.9%), and albumin exhibited the highest specificity (97.3%) [19].

Our study is among the few that have investigated the relationship between hypoalbuminemia and clinical and biological severity scores. We found a significant association with persistent SIRS > 48 h (*P* < 0.001) and a BISAP score ≥ 3 (*P* < 0.001). However, ours is the only study that has examined the association with CTSI, which was also found to be significant (*P* = 0.001).

Similarly to our study, a retrospective study involving 1000 patients revealed significant differences in the hypoalbuminemia group compared to the normal group. The differences included hematocrit (*P* = 0.023), serum creatinine (*P* < 0.001), and APACHE II score ≥ 15 (17.6% vs. 23.4%, *P* < 0.001). However, no significant association was found for SIRS [5].

The strengths of our study also include being the first to investigate the relationship between hypoalbuminemia and the occurrence of local complications. A significant association was found with the development of WON. However, intriguingly, no significant association was observed with the occurrence of pseudocysts. The varying significance of our findings between WON and pseudocyst underscores the importance of considering the underlying pathophysiological differences between these pancreatic conditions. In WON, the presence of necrotic tissue and the associated inflammatory response likely play a key role in determining the clinical relevance of our results. The heightened inflammatory milieu in WON, characterized by elevated levels of pro-inflammatory cytokines and infiltrating immune cells, may amplify the impact of the factors we identified compared to the more quiescent inflammatory state typically seen in pseudocyst.

A significant focus in the current literature is on exploring the relationship between albumin infusions and the prognosis of critically ill patients. In a meta-analysis, a comparison with crystalloid revealed a trend towards reduced 90-day mortality in severe sepsis patients who underwent resuscitation with albumin (OR: 0.88; 95% CI, 0.76 to 1.01; *P* = 0.08). Notably, the use of albumin for resuscitation significantly decreased 90-day mortality in septic shock patients (OR 0.81; 95% CI, 0.67 to 0.97; *P* = 0.03) [20]. The ALBIOS study reported that albumin infusion in septic shock patients was associated with a decrease in mortality (RR: 0.87, 95% CI: 0.77–0.99).

In the context of AP, a few studies have explored whether albumin administration confers a beneficial impact on the outcomes. Huiting Xu and al demonstrated a lower mortality rate (OR: 0.52, 95% CI: 0.29–0.92, *P* = 0.023) among hypoalbuminemia patients who received albumin infusion compared to those who did not [5]. However, in a retrospective study involving a total of 950 patients, albumin infusion did not confer benefits in terms of patients’ 28-day or Intensive Care Unit (ICU) mortality. Intriguingly, it was significantly associated with an extended duration of both hospital and ICU stays [21].

Several limitations should be noted in the current study. Firstly, it is a retrospective cohort study, which may introduce selection bias. Additionally, the data were collected exclusively from a single tertiary care hospital, and the sample size is somewhat limited. Moreover, the study only includes a single-time measurement of albumin and lacks data on sepsis.

## Conclusion

The study provides evidence for the significance of serum albumin as a prognostic biomarker in AP, especially in predicting sustained organ failure and mortality. This insight has the potential to improve the care and outcomes of individuals with AP. Nevertheless, further researches are crucial to validate these findings and investigate the potential advantages of incorporating serum albumin levels into clinical decision-making for AP.

## Data Availability

The data supporting the findings of this study are available from Mohammed VI University Hospital in Oujda, but restrictions apply to the availability of these data, which were used under license for the current study and so are not publicly available. Data are however available from the authors upon reasonable request and with permission of Mohammed VI University Hospital in Oujda.
